# Screening of sleep apnea based on heart rate variability and long short-term memory

**DOI:** 10.1007/s11325-020-02249-0

**Published:** 2021-01-10

**Authors:** Ayako Iwasaki, Chikao Nakayama, Koichi Fujiwara, Yukiyoshi Sumi, Masahiro Matsuo, Manabu Kano, Hiroshi Kadotani

**Affiliations:** 1 Faculty of Medicine, Kyoto University, Kyoto, Japan; 2 Department of Systems Science, Kyoto University, Kyoto, Japan; 3 Department of Material Process Engineering, Nagoya University, Furo-Cho, Chikusa-Ku, Nagoya, 464-8601 Japan; 4 Department of Psychiatry, Shiga University of Medical Science, Otsu, Japan; 5 Department of Sleep and Behavioral Sciences, Shiga University of Medical Science, Otsu, Japan

**Keywords:** Sleep apnea syndrome, Wearable sensor, Wearable sensor, Machine learning, Telemedicine

## Abstract

**Purpose:**

Sleep apnea syndrome (SAS) is a prevalent sleep disorder in which apnea and hypopnea occur frequently during sleep and result in increase of the risk of lifestyle-related disease development as well as daytime sleepiness. Although SAS is a common sleep disorder, most patients remain undiagnosed because the gold standard test polysomnography (PSG), is high-cost and unavailable in many hospitals. Thus, an SAS screening system that can be used easily at home is needed.

**Methods:**

Apnea during sleep affects changes in the autonomic nervous function, which causes fluctuation of the heart rate. In this study, we propose a new SAS screening method that combines heart rate measurement and long short-term memory (LSTM) which is a type of recurrent neural network (RNN). We analyzed the data of intervals between adjacent R waves (R-R interval; RRI) on the electrocardiogram (ECG) records, and used an LSTM model whose inputs are the RRI data is trained to discriminate the respiratory condition during sleep.

**Results:**

The application of the proposed method to clinical data showed that it distinguished between patients with moderate-to-severe SAS with a sensitivity of 100% and specificity of 100%, results which are superior to any other existing SAS screening methods.

**Conclusion:**

Since the RRI data can be easily measured by means of wearable heart rate sensors, our method may prove to be useful as an SAS screening system at home.

**Supplementary Information:**

The online version contains supplementary material available at (10.1007/s11325-020-02249-0)

## Introduction

Sleep apnea syndrome (SAS) is a disorder in which apnea and hypopnea occur frequently during sleep. The severity of SAS is defined by the number of apnea and hypopnea per hour, which is called apnea hypopnea index (AHI), and people whose AHI is more than of 15 are defined as having moderate-to-severe SAS. There are three types of sleep apnea: obstructive sleep apnea (OSA), central sleep apnea (CSA), and a combination of the two.

Patients with SAS suffer from daytime sleepiness as well as increased risk in lifestyle-related diseases [1–3]. Although SAS is a prevalent disorder, 80–90% of patients are undiagnosed and untreated [4]. This is partly because the gold standard test, polysomnography (PSG), requires special equipment and experts, limiting the number of facilities that can perform PSG. Thus, portable monitoring devices are used for SAS screening [5]. Although these devices can be used at home, they require operational skills and their screening accuracies are not sufficiently high [6]. Therefore, a high-performance SAS screening system that can be used easily at home is needed.

There are two major ECG-based apnea detection methods–ECG-derived respiratory signal (EDR) and heart rate variability (HRV) [7–10]. EDR is fluctuation of thoracic impedance derived when respiratory movement occurs. This leads to a shift in the electric axis and morphological changes in the ECG signal. Varon et al. reported that the sensitivity and the specificity of their EDR-based apnea screening algorithm were both 84% [11]. However, there is a technical difficulty in detecting all P – T waves accurately. In addition, ECG measurement devices for medical purposes, such as the Holter monitor, are expensive and difficult to handle for non-healthcare professionals,v which make these methods inapplicable for home-use SAS screening.

In patients with apnea, changes in respiratory conditions significantly affect electrocardiogram (ECG) data, and this can be utilized in the detection of apnea. Heart rate variability (HRV) is an ECG-based apnea detection method, defined as changes in intervals between R waves (R-R interval; RRI). HRV is a useful measure to quantify changes in activities of the autonomic nervous system used in many fields. Apnea during sleep occurs due to a change in the autonomic nervous function as a result of a decrease in saturation of peripheral oxygen (SpO_2_), causing changes in HRV [12]. Because RRI data can be collected precisely using inexpensive and simple wearable devices [13], the use of HRV for apnea screening is more suitable than EDR from a practical point of view. Typical HRV features include time-domain features like mean or variance of RRI, or frequency domain features like power spectral density (PSD) [14]. For example, Nakayama et al. used these HRV features and discriminated between apnea or normal respiration by means of a random forest model, achieving a sensitivity of 76% and a specificity of 92% [10]. This performance should be improved for future clinical application.

In order to realize precise apnea screening, we propose a new screening method that utilizes RRI and machine learning. In the method, the raw RRI data measured during sleep are split into multiple segments, and each piece of segmented RRI data is discriminated between apnea or normal respiration by means of a machine learning model. We use long short-term memory (LSTM) for this discrimination, which is a type of neural network model. Based on algorithms preliminarily reported by [15, 16], the present study examined the utility and performance of the model by applying the algorithm to a wider range of data including real-life clinical data.

## Method

In this section, we propose a new method for screening SAS by combining RRI measurement and LSTM.

An overview of the proposed SAS screening method is as follows: A series of RRI derived from a subject is split into multiple segments of a fixed length, and each segment is determined to be either normal respiration or apnea. Finally, the ratio of the period classified as “apnea” to the total sleep period (apnea/sleep ratio; AS ratio) [10] is calculated, and the subject is regarded as a “potential patient” if his or her AS ratio is greater than a predefined threshold.

Before describing the proposed SAS screening method, LSTM is explained briefly.

### Long short-term memory

**Fig. 1:**
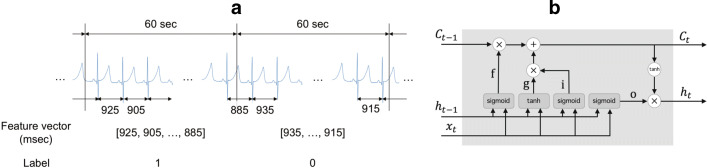
**a** Feature extraction framework. **b** LSTM architecture. sigmoid: sigmoid function $\sigma (x) = \frac {1}{1 + \exp (-x)}$, ⊕: calculate the sum of two matrices, ⊗: calulate the hadamard product of two matrices

In a feed-forward neural network (FNN), the direction of information flow is from the input layer to the output layer only, and information never propagates in the backward direction [17]. On the other hand, a recurrent neural network (RNN) receives the output of the previous time point, hidden state *h*, as input in addition to the current measurements. This feature of RNN enables the handling of time-series data; however, simple RNN cannot learn long-term dependency. The long-term dependency problem can be solved using a modified version of RNN called long short-term memory (LSTM). Figure 1b shows an internal state of LSTM [18] at time point *t*. LSTM has a cell memory *C*_*t*_, which can store long-term memories. That is, LSTM reads, writes, and resets the long-term memories through an input gate (i), an output gate (o), and a forget gate (f), respectively. The input and output gates control the flow of the input and the output of the memory cell activation, and the forget gate serves to reset memory cells. By introducing memory cells and gates, LSTM can deal with exploding or vanishing gradient problems as well as long-term dependency [18].

### Model training

Algorithm 1 is adopted for LSTM model training. A series of the raw RRI collected during sleep is described as $\boldsymbol {z}^{\{i\}} = [z^{\{i\}}_{1}, ..., z^{\{i\}}_{t}, ...]^{T}$ (*i* = 1,...,*I*), where $z^{\{i\}}_{t}$ is the *t* th RRI extracted from the *i* th subject and *I* denotes the total number of subjects in the training dataset. In order to handle individuality, ***z***^{*i*}^ was standardized to $\boldsymbol {\tilde {z}}^{\{i\}}$, which has zero mean and unit variance.

In step 4, $\boldsymbol {\tilde {z}}^{\{i\}}$ is divided into periods of 60 s. The RRI segmentation framework is illustrated in Fig. 1 (a). $\boldsymbol {x}^{\{i\}}_{n} = [\tilde {z}^{\{i\}}_{k_{n}}, ..., \tilde {z}^{\{i\}}_{j_{n}}]^{T}$, where $k_{n} \in \mathfrak {N}$ satisfies ${\sum }_{t=1}^{k_{n} - 1} z^{\{i\}}_{t} {\leq } 60(n - 1) {\leq } {\sum }_{t=1}^{k_{n}} z^{\{i\}}_{t}$ and $j_{n} \in \mathfrak {N}$ satisfies ${\sum }_{t=1}^{l_{n}} z^{\{i\}}_{t} {\leq } 60 n {\leq } {\sum }_{t=1}^{l_{n} + 1} z^{\{i\}}_{t}$, is used as a feature vector.

Experts classify each ***x***^{*i*}^ into apnea $\mathcal {A}$ or normal respiration $\mathcal {N}$ based on the PSG data, and a label vector collected from the *i* th subject is described as $\boldsymbol {y}^{\{i\}} = [y^{\{i\}}_{1}, ..., y^{\{i\}}_{n}, ...]^{T}$, where $y^{\{i\}}_{n} = \{\mathcal {A}, \mathcal {N}\}$.

Next, feature vectors are arranged as ***X***^{*i*}^ whose *n* th row is $\boldsymbol {x}^{\{i\}}_{n}$, which are then combined to produce a matrix ***X*** as follows.

$$\boldsymbol{X} = \begin{bmatrix} \boldsymbol{X}^{\{1\}} \\ \boldsymbol{X}^{\{2\}} \\ {\vdots}   \\ \boldsymbol{X}^{\{I\}} \end{bmatrix} .$$

In step 7, ***y***^{1}^,...,***y***^{*I*}^ are concatenated lengthwise to give a vector ***y***.

$$\boldsymbol{y} = \begin{bmatrix} \boldsymbol{y}^{\{1\}} \\ \boldsymbol{y}^{\{2\}} \\ {\vdots}   \\ \boldsymbol{y}^{\{I\}} \end{bmatrix} .$$

Finally, the LSTM model *m*(⋅) that distinguishes apnea from normal respiration is trained from ***X*** and ***y***.

### Apnea screening procedure

Algorithm 2 describes a procedure for determining whether or not a subject has apnea. In step 2, the raw RRI data ***z*** are collected during sleep. ***z*** is standardized to $\boldsymbol {\tilde {z}}$, which is then split into periods of 60 s to create ***x***_*n*_.

The model classifies the respiratory condition of every segmented RRI data into normal or apnea. The model is written as $\boldsymbol {\hat {y}}_{n} = m(\boldsymbol {x}_{n})$ where *m*(⋅) is a function and $\boldsymbol {\hat {y}}_{n}$ is an estimated respiratory condition $\boldsymbol {\hat {y}}_{n} = {\{\mathcal {A}, \mathcal {N}\}}$ corresponding to ***x***_*n*_.

In order to classify a subject as a patient with moderate-to-severe SAS or a healthy person, the apnea/sleep (AS) ratio *A* is defined as follows:

$$ A = 100 \times T_{a} / T_{s} ~ [<percent>] $$

where *T*_*s*_ and *T*_*a*_ are the total sleep time (TST) and the sum of apnea periods determined by the model *m*(⋅), respectively. The subject is regarded as a “potential patient with moderate-to-severe SAS” if *A* is more than a predefined threshold $\bar {A}$, and otherwise as a “healthy person.”

### Data description

The PSG data from patients and healthy persons were collected at the Shiga University of Medical Science (SUMS) hospital. The inclusion criteria were the following: aged 18 or more; absence of hypertension, diabetes, heart failure, angina, myocardial infarction, arrhythmia, and depression. PSG recording during sleep (6–7 h) was conducted in an EEG recording shield room in the presence of sleep specialists. A PSG system (Alice 5, Philips) included video, EEG, ECG (lead II, sampling frequency: 200Hz), EOG, EMG, SpO_2_, chest and abdominal wall movements for respiratory efforts, nasal airflow, and thermistor for respiratory monitoring. PSG data with strong artifacts in the ECG data were removed, and apnea or normal respiration were labeled, based on the PSG data, by Polysomnographic Technologists as certified by the Japanese Society of Sleep Research. Subjects were classified into patients with moderate-to-severe SAS (AHI ≥ 15) or healthy subjects (AHI < 15). The average and standard deviation of AHI was 38.2 and 18.2 in the modeling dataset, and 40.4 and 18.0 in the validation dataset. The summary of subject profiles is shown in Table 1, and the detailed profile of each subject is shown in Supplemental Table S.1.

**Table 1:** Subject profile

	Male			Female		
Age	AHI 0–14	15–29	30–	0–14	15–29	30–
18–30	7	0	0	15	0	1
31–50	7	2	5	6	0	0
51–80	0	7	7	0	1	1

**Fig. 2:**
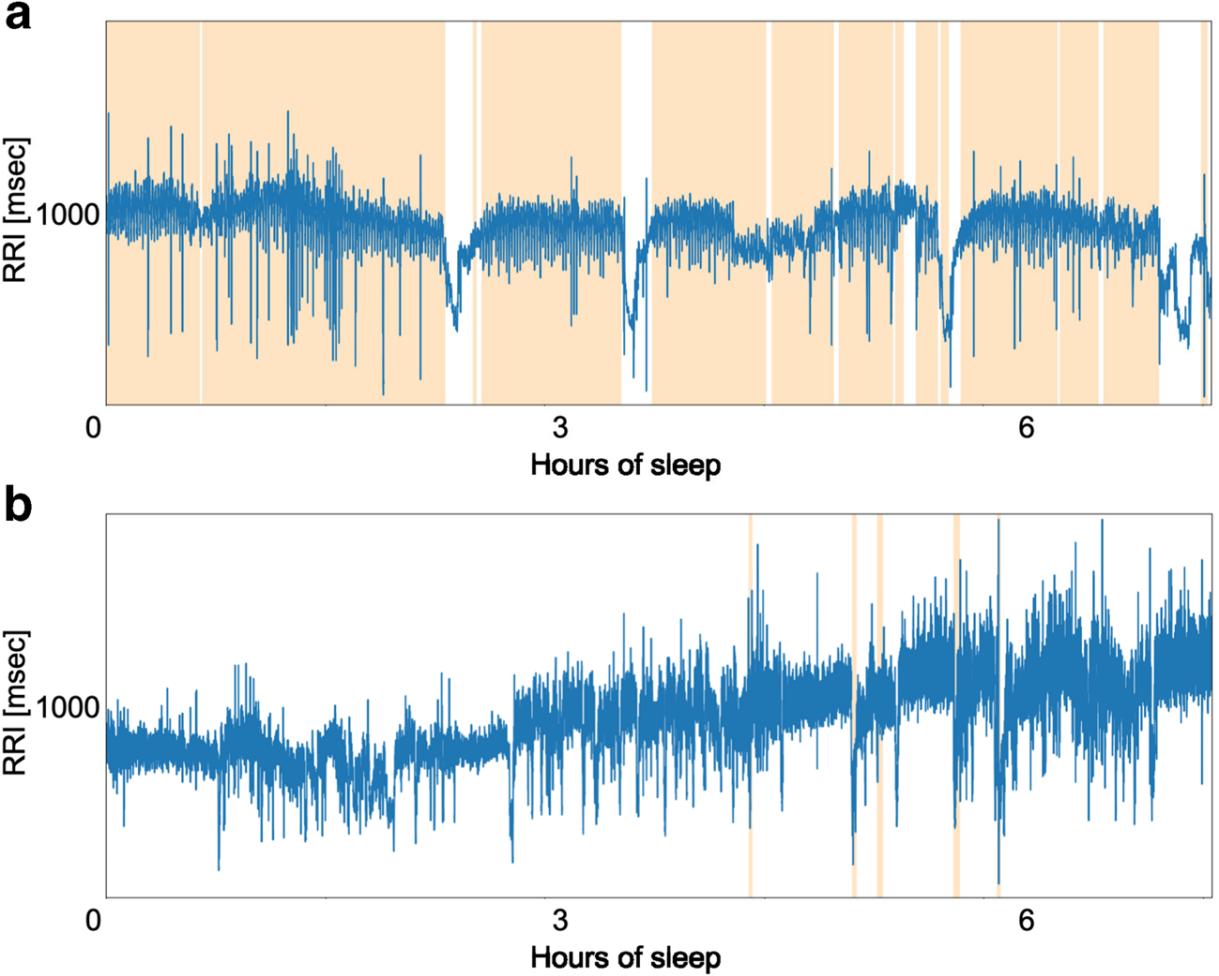
**a**, **b** Example of RRI: patient P1 (top) and healthy person H2 (bottom)

Patients P1–P23 had OSA, while only patient P24 had CSA. The ECG data were extracted from the PSG data, and R waves were detected using the Pan-Tompkins algorithm [19]. Figure 2 shows RRI data collected from patient P1 (male, 76 years old, AHI = 56.9) and healthy subject H2 (female, 19 years old, AHI = 0), where apnea periods are illustrated with colored bands. These data indicate that there were more evident fluctuations of RRI during apnea compared to during normal respiration.

Finally, we divided all clinical data into a training dataset (P1–P13 and H1–H18) and a validation dataset (P14–P24 and H19–H35).

### Statistical analysis

We used the Welch’s *t* test for comparison of the estimated AS ratios between the patients and the healthy subjects, and its significance level was set to *p* < 0.05. Computation in this study was performed in Python 3.6.6 with SciPy 1.1.0, and TensorFlow 1.10.0.

## Results

In this section, we report the results of applying the proposed SAS screening method to clinical data.

### Model training

In LSTM training, 5-fold cross-validation was conducted using a training dataset in order to tune hyperparameters. We chose a network with an LSTM layer with 32 units in the hidden layer, trained for 150 epochs with the Adam optimizer, whose learning rate was 0.01.

Patient P5 (female, 66 years old, AHI = 15.3) in the training dataset, whose AHI was the smallest among apnea patients in the training dataset, was defined as a borderline case to discriminate healthy persons from patients with moderate-to-severe SAS, and her AS ratio (0.168) was used as the threshold $\bar {A}$.

### Screening results

**Fig. 3:**
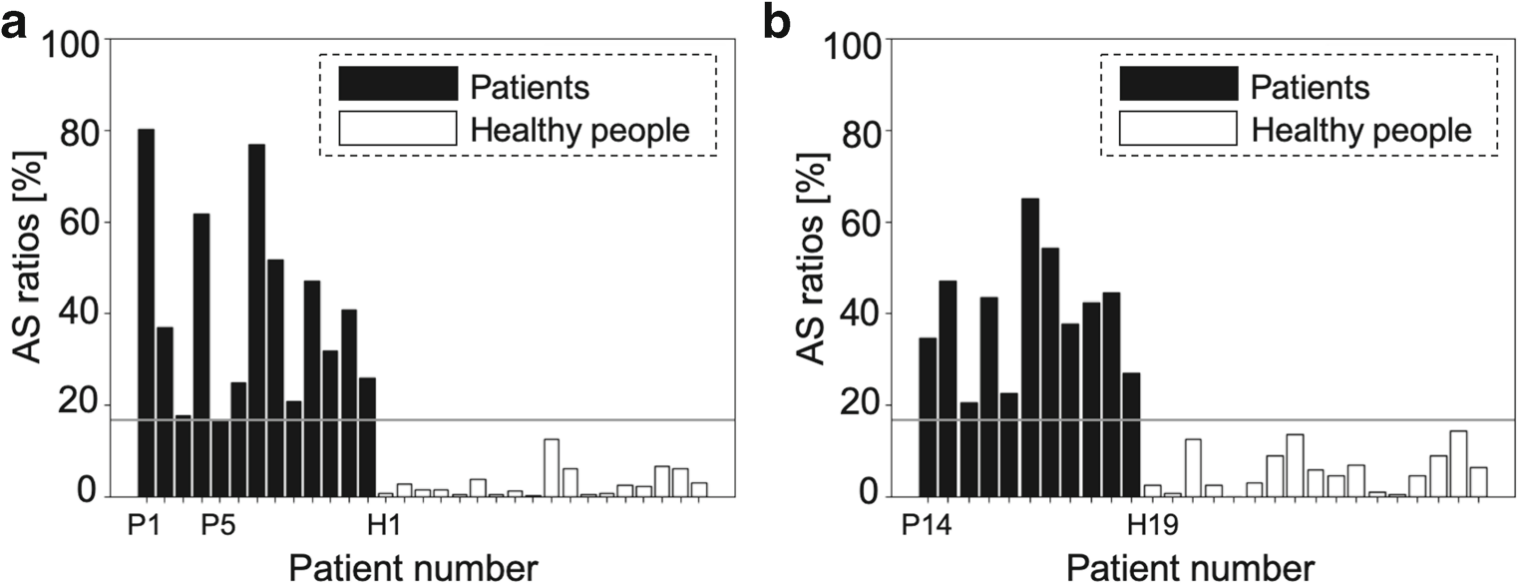
**a**, **b** AS ratios when using raw RRI: modeling data (left) and validation data (right)

The screening result of the proposed method is illustrated in Fig. 3, where each bar represents the AS ratio for each subject in the modeling dataset (a) and validation dataset (b). As shown in Fig. 3b, the proposed method achieved a sensitivity of 100% and a specificity of 100%. The means of AS ratios of the patients and healthy persons in the validation dataset were 0.933 and 0.058, respectively, which resulted in statistical significance (*p*< 0.01).

## Discussion

The proposed SAS screening algorithm achieved a sensitivity of 100% and a specificity of 100% in the validation dataset. Table 2 shows the accuracies of portable monitoring devices [20] and the proposed method, which shows that the performance of the proposed method was higher than that of existing portable monitoring devices.

**Table 2:** Screening accuracy of existing devices and the proposed algorithm

Product	Sensitivity (%)	Specificity (%)
Healthdyne 202-11 Oximeter	97	80
Nellcor N-200	82	76
SageTech SNORESAT	100	63
ResMed AutoSet 3.03	97	32
Criticare 504 5 0ximeter	67	92
Konica Minolta Pulsox 7	94	62
Proposed	100	100

To confirm the robustness of our model, we performed the following two additional experiments. In the first experiment, we rearranged the modeling data and validation data at random five times, which resulted in an average AUC (area under the curve) of 0.96. In addition, we applied our algorithm to the open dataset (PhysioNet Apnea-ECG Database [21], *N* = 35), which resulted in an average AUC of 0.95. These results showed that the performance of our model does not greatly deteriorate when we change the modeling and the validation data, which suggests that our model is robust enough to maintain good performance despite the small sample size.

In addition, the correlation coefficients between the AS ratio and sleep parameters (arousal index, sleep efficacy, wake time after sleep onset; WASO, oxygen desaturation index; ODI, average SpO_2_ during sleep) were 0.71, − 0.52, 0.64, 0.67, and − 0.65, respectively. That is, the AS ratio is positively correlated with Arousal index, WASO, and ODI, and negatively correlated with sleep efficacy and average SpO_2_. Thus, our methodology can calculate a reliable parameter of the quality of sleep using only single channel RRI data, which is an advantage of the proposed method. However, the correlation coefficient between the AS ratio and AHI was 0.76, which is not high enough for severity diagnosis. Thus, it is difficult to estimate the severity of SAS by means of the proposed method.

**Fig. 4:**
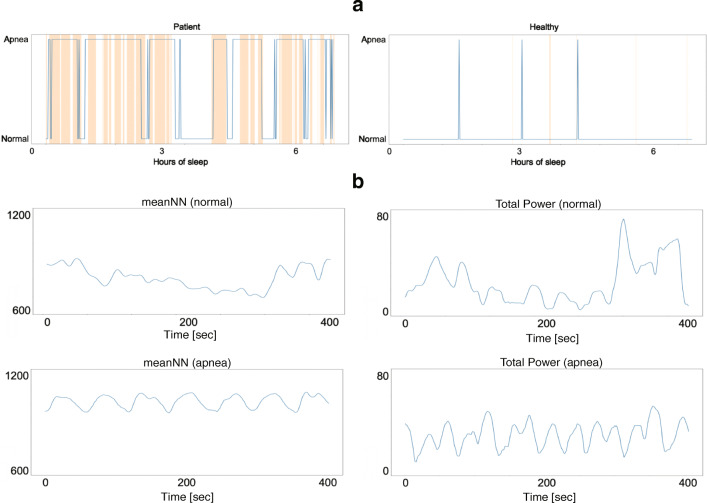
**a** Example of screening result for each subject: a patient (left) and a healthy subject (right). **b** CVPR of patient P1 (top: normal respiration period, bottom: apnea period)

Figure 4a shows examples of discrimination results of patient P21 (male, 58 years old, AHI = 31.7) and healthy person H20 (female, 24 years old, AHI = 0) in the validation dataset. The orange-colored bands denote the apnea periods, and the blue line is the respiratory condition determined by the LSTM model. The periods discriminated as “apnea” start before the apnea onset and continue even after apnea ends. This result is consistent with a previous report that HRV changes not only during apnea periods but also before and after them [22].

In order to confirm the effect of the window width for RRI segmentation on the screening performance, we changed the window width to 30 s, 60 s, and 120 s: a sensitivity of 91% and a specificity of 76% were achieved in the 30-s window width, and a sensitivity of 82% and a specificity of 88% were achieved in the 120-s window width. Thus, the screening performances in the 30-s and the 120-s window widths were worse than that in the 60-s window width (a sensitivity of 100% and a specificity of 100%), which indicates that a window width of 60 s is appropriate for RRI segmentation. This might be because the time period of 60 s corresponds to the periodic time of cyclical variation of heart rate (CVHR) [23]. Figure 4b shows time-domain HRV features (meanNN and Total Power) of the RRI of patient P1 (male, 76 years old, AHI = 56.9). These figures showed periodic changes in HRV when apnea occurred, and its period was about 60 s, which is called CVHR. Thus, it is concluded that the window width of 60 s for RRI segmentation may allow extraction of specific characteristics of the RRI data during apnea.

**Fig. 5:**
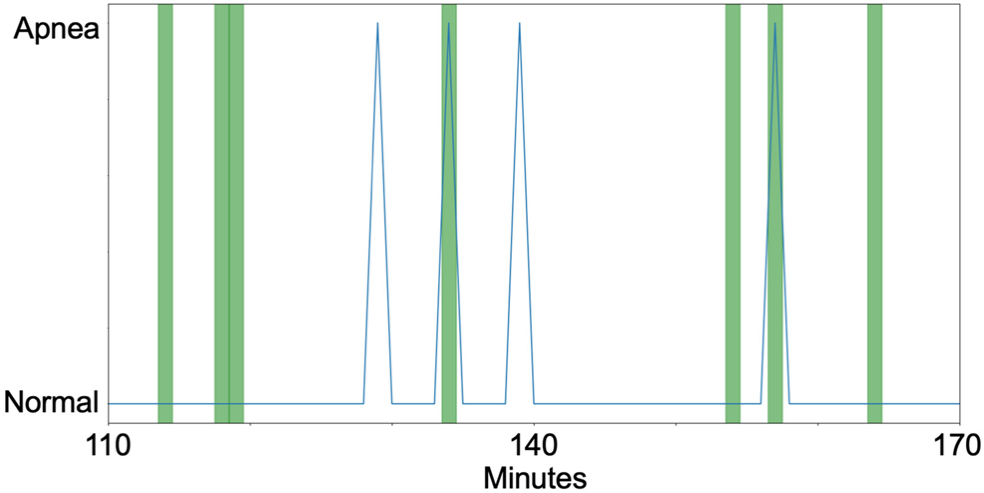
Arrhythmic events and classification results: arrhythmia period (green) and estimated respiratory conditions (blue)

Since our method uses raw RRI data, the screening result of the proposed method may be significantly affected by arrhythmia. Some studies have shown that patients with arrhythmia show changes in RRI regardless of apneic events [24], which might cause misclassification. Figure 5 shows an arrhythmia episode and its apnea discrimination result of subject H21 (male, 23 years old, AHI = 0.4) in the apnea-free period, where the green-colored bands denote arrhythmia periods. These data suggest that arrhythmia can be misclassified as apnea, which might be resolved by adding additional data from patients with arrhythmia in the training dataset.

Nakayama et al. [10] proposed a method that combines HRV features extracted from the RRI data and random forest, which discriminated subjects with a sensitivity of 76% and a specificity of 92%. The Nakayama method and the proposed method is different in two points, input features (raw RRI vs HRV features) and the machine learning algorithm (LSTM vs random forest). In order to investigate differences in the performance between the Nakayama method and the proposed method, we additionally tried an LSTM model with HRV features instead of the raw RRI.

**Fig. 6:**
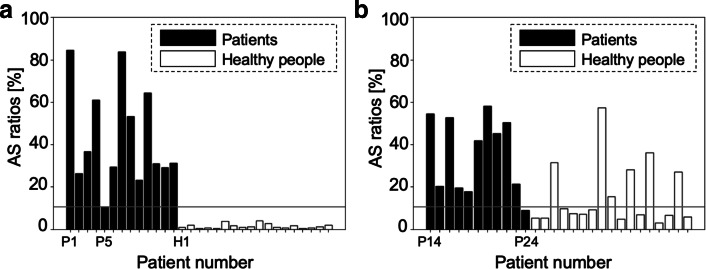
**a**, **b** AS ratios when using HRV features: modeling data (left) and validation data (right)

We used eleven HRV features (meanNN, SDNN, Total Power, RMSSD, NN50, pNN50, LF, HF, LF/HF, LFnu, HFnu) following [10] and the HRV analysis guideline [14]. We trained the LSTM model from HRV features as input to classify each period as apnea or normal respiration. Fivefold cross-validation was conducted using a training dataset to tune the hyperparameter, and a network with an LSTM layer with 32 units in the hidden layer was constructed, which was trained for 150 epochs with the Adam optimizer whose learning rate was 0.01. Patient P5 (female, 66 yearls old, AHI = 15.3), whose AHI was the smallest among patients with moderate-to-severe SAS in the training dataset, was defined as a borderline case to distinguish patients with moderate-to-severe SAS from others. Her AS ratio (0.168) was used as the threshold $\bar {A}$. The result is described in Fig. 6, which shows that the sensitivity and the specificity in the validation dataset were 91% and 64%, respectively.

HRV feature extraction requires RRI data of at least 2 min according to the guideline [14]. Since the period of CVHR is about 60 s, the changing cycle of the heart rate during apnea is shorter than the period required for HRV feature extraction, which may lead to loss of information when the raw RRI data are converted to HRV features. On the other hand, the use of the raw RRI data in the proposed algorithm enabled the detection of high time-resolution characteristics such as rapid RRI changes, which is not feasible with normal HRV analysis. RNN including LSTM can handle time series information appropriately unlike FNN; thus, the combination of raw RRI and LSTM achieved high screening performance.

In addition, there is the possibility that the proposed method can deal with CSA as well as OSA. Although patient P24 (female, 23 years old, AHI = 75.8), who had CSA, was screened correctly by means of the proposed method, she was misclassified as healthy when HRV features were used as inputs of the LSTM model. Ref. [25] showed that patients with CSA have more regular patterns of CVHR than patients with OSA, which indicates that CSA can be easily distinguished from normal respiration. Thus, patients with CSA may be screened with high sensitivity; however, we need further investigation because we have only one CSA patient in our clinical data.

In order to realize a home-use SAS screening device by utilizing the proposed method, sensors that can measure ECG are required. Several wearable heart rate measurement devices have been proposed. For example, ref. [13] developed a wearable sensor that measures RRI easily and precisely. By combining these wearable sensors and the proposed method, a home-use SAS screening system could be realized.

The limitation of this work includes the clinical data; all subjects in this experiment are Japanese. In addition, patients with diseases that may affect HRV were excluded from the data. Additionally, it was difficult to investigate the effect of age distribution on learning due to the limited number of subjects. Thus, we need to collect further HRV data from persons with various backgrounds and confirm the accuracy of our method.

## Conclusion

In this research, we proposed an SAS screening algorithm that uses raw RRI data and LSTM. The LSTM model determines whether or not a person has apnea based on the raw RRI data during sleep. When we applied our algorithm to the clinical data, patients with apnea were screened perfectly; that is, the sensitivity and the specificity were both 100%.

The clinical data used in this research were extracted from the PSG recordings, and sleep onset was determined based on the EEG data. In order to realize a home-use SAS screening system based on the proposed method, an automatic sleep onset detection method based on ECG data is needed. Since a recent study has proposed a sleep stage classification method using ECG data [26], an SAS screening system that depends solely on the ECG data could be realized by combining the proposed method and the ECG-based sleep stage classification method.

In future works, we will apply the proposed algorithm to other clinical PSG datasets, including open data, to validate its performance. Although we trained and validated our model on a desktop PC in this research, we will implement our algorithm on a smartphone app that can connect to wearable heart rate sensors in order to realize the home-use SAS screening system. 
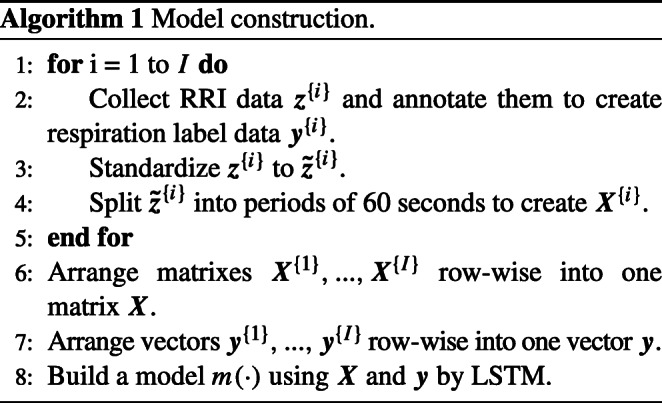

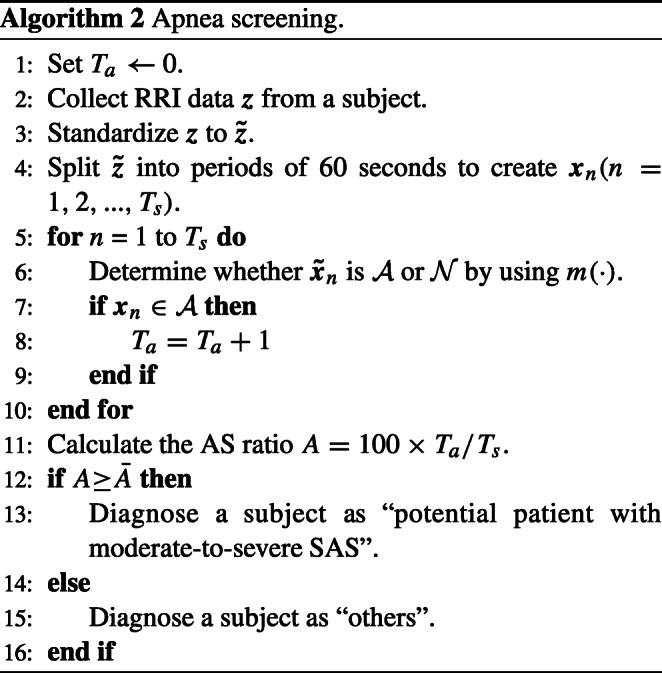


## Electronic supplementary material

Below is the link to the electronic supplementary material.

(PDF 7.50 KB)

## Data Availability

The PSG data will be made available by the corresponding author to colleagues who propose a reasonable scientific request after approval by the institutional review board of the SUMS Hospital. The source code developed in this study will be made available by the corresponding author to colleagues who propose a reasonable scientific request.
