# Supplementary material for: Screening of sleep apnea based on heart rate variability and long short-term memory
Source: Sleep Breath. 2021 Jan 10;25(4):1821–9. doi: 10.1007/s11325-020-02249-0 (PMC8590683; doi:10.1007/s11325-020-02249-0)
Supplement: Supplementary file 1 — (PDF 7.50 KB) [file 11325_2020_2249_MOESM1_ESM.pdf]

Table 1: Profiles of subjects included in this study

Subject	Sex	Age	BMI	AHI	Subject	Sex	Age	BMI	AHI
H1	F	19	20.5	1.2	H31	F	46	22.1	6.5
H2	F	19	18.3	0.0	H32	F	31	21.6	2.1
H3	F	20	21.7	0.0	H33	F	46	23.8	1.6
H4	F	20	21.9	0.6	H34	M	38	28.7	0.9
H5	F	19	26.6	0.8	H35	M	36	22.2	11.8
H6	M	19	24.1	0.6	P1	M	76	21.7	56.9
H7	F	23	19.2	0.3	P2	M	58	27.4	16.7
H8	M	23	21.5	1.5	P3	M	69	23.0	19.9
H9	M	24	23.1	0.6	P4	M	45	29.0	25.6
H10	M	42	24.9	0.7	P5	F	66	25.8	15.3
H11	M	42	19.6	3.1	P6	M	63	26.4	25.8
H12	M	23	19.8	1.5	P7	M	55	29.8	63.2
H13	F	40	22.2	0.7	P8	M	71	24.7	40.8
H14	F	40	21.6	0.6	P9	M	51	23.3	16.1
H15	F	23	19.7	1.8	P10	M	51	33.1	44.7
H16	M	25	27.2	6.5	P11	M	46	29.6	21.9
H17	F	24	25.6	0.0	P12	M	40	24.8	32.1
H18	F	19	20.3	0.2	P13	M	28	18.4	30.8
H19	F	21	18.8	0.0	P14	M	65	27.2	20.1
H20	F	24	20.5	0.0	P15	M	51	23.7	26.8
H21	M	23	20.8	0.4	P16	M	72	24.9	38.6
H22	M	19	19.2	0.2	P17	M	77	22.1	28.1
H23	F	19	20.3	0.0	P18	M	42	38.2	49.3
H24	F	19	20.3	0.0	P19	M	76	28.3	52.1
H25	M	45	23.1	7.9	P20	M	49	27.2	44.0
H26	M	33	23.4	1.9	P21	M	58	23.6	31.7
H27	F	23	19.5	0.3	P22	M	37	30.0	58.9
H28	F	37	18.7	0.0	P23	F	68	26.8	30.7
H29	M	31	21.7	1.4	P24	F	23	31.5	75.8
H30	F	22	19.2	1.8					
